# Analysis of pulsatile motion in the murine cornea using phase-sensitive visible-light optical coherence tomography

**DOI:** 10.1364/BOE.565471

**Published:** 2025-08-08

**Authors:** Lucas May, Sybren Worm, Maria Varaka, Georg Ladurner, Yash Patel, Antonia Lichtenegger, Sarah Nagl, Andreas Drauschke, Conrad Merkle, Ireneusz Grulkowski, Bernhard Baumann

**Affiliations:** 1 Center for Medical Physics and Biomedical Engineering, Medical University of Vienna Währinger Gürtel 18-20/4L, 1090 Vienna, Austria; 2 Department of Life Science Engineering, UAS Technikum Wien, Höchstädtplatz 6, 1200 Vienna, Austria; 3 Institute of Physics, Faculty of Physics, Astronomy and Informatics, Nicolaus Copernicus University, ul. Grudziądzka 5, 87-100 Toruń, Poland; 4 Institute of Biomedical Physics, Medical University of Innsbruck, Müllerstraße 44, 6020 Innsbruck, Austria

## Abstract

The cornea is constantly exposed to cardio-pulmonary induced changes of intraocular pressure. Resulting pulsatile motion patterns in the eye may indicate severe pathological alterations of the biomechanical composition and the physiological function in affected tissue types. Non-contact measurements of pulsatile motion in the anterior eye could thus hold a diagnostic value, not only to assess biomechanics in corneal pathologies, but also pulsatile irregularities, resulting from the stimulus itself. Here, we present a methodology to measure physiologically induced tissue compression in the central murine cornea, based on a prototype for visible light optical coherence tomography (vis-OCT) providing 2-µm axial resolution and phase-related nanoscale measurements of displacement inside the corneal tissue. We present the in vivo assessment of pulsatile deformations in the cornea (passive stimulation) as well as ex vivo investigations of tissue oscillations induced by active stimulation using an air puff in the eyes of wild-type mice. The frequency spectra of corneal tissue oscillations were measured and showed good agreement with physiological control measures reported in the literature. Based on these preliminary results, revealing physiologically induced compression and air-puff induced nano-oscillations in the murine cornea, the methods may be extended towards optical coherence elastography in the visible range, to access biomechanical parameters, complementing the high-resolution structural imaging capabilities of vis-OCT.

## 1. Introduction

Tissue motion in the eye, whether naturally occurring as the ocular pulse, or in form of actively induced oscillations, has been found to be influenced by various factors, such as the morphological shape, the intraocular pressure (IOP), the biomechanical tissue composition or the cardiovascular stimulation patters, originating from the back of the eye [1–3]. Among these, the elasticity of the cornea plays a particularly critical role in understanding ocular diseases such as keratoconus, and in evaluating the outcomes of refractive surgeries [4–7]. Analyzing motion or displacement patterns within the corneal tissue may therefore provide insights into pathological changes in tissue elasticity or other related biomechanical parameters. By exploiting the phase-sensitive character of optical coherence tomography (OCT), such motion in the tissue can be tracked with a sensitivity down to the sub-nanometer range [8].

Building on these capabilities, optical coherence elastography (OCE) has emerged as a powerful tool for measuring the mechanical properties of tissues in a non-invasive manner, with potential relevance in ophthalmology for the non-invasive assessment of corneal biomechanics [9]. In conjunction with mechanical models, these measurements allow for the reconstruction and absolute quantification of elastic tissue properties, such as the shear wave velocity, natural frequency or the Young’s modulus [10,11]. While most OCE methods rely on external mechanical stimulation to induce detectable tissue displacements [10–12], recent advancements have explored passive elastography approaches [13–17]. Similar to Doppler or OCTA, these methods utilize intrinsic physiological motion, such as heartbeat or pulsatile blood flow, and integrate it as natural stimulation sources to probe tissue mechanics without the need for external excitation. In terms of probing the mechanical properties in the cornea, omitting the external stimulation could lead to improvements in patient comfort and reduce the technical complexity of the OCE system. However, missing information about the stimulus characteristics, including applied forces and its propagation characteristics through different tissue types, obstruct the capability of an absolute measurement of elasticity via passive OCE *in vivo*. Thus, most of the research so far, focused on the development of passive OCE approaches primarily as an additional visualization channel of physiological tissue motion and an imaging extension, which serves as an indicator of relative elasticity in response to the physiological stimulation. Regardless of the origin of the stimulus, taking into account the functional characteristics of OCE in general, a combination with high-resolution structural OCT could provide valuable complementary information about the progression of corneal pathologies e.g., in order to study early-stage structure-function relationships.

A suitable approach considers the use of visible light for optical coherence tomography (vis-OCT) to achieve higher axial resolution and enhanced contrast in superficial tissues, when compared to systems working in the near-infrared (NIR) range [18]. Previous studies have demonstrated the potential of vis-OCT, ranging from high-resolution structural imaging and oxygen mapping in the retina, to *ex vivo* investigation of Alzheimer’s pathology [19–21]. The cornea, although being highly transparent, exhibits sufficient scattering at visible wavelengths and is particularly well suited for imaging in this spectral range, where shorter wavelengths enhance light–tissue interactions and improve contrast [22]. Theoretically, increased scattering coefficients may also contribute to improved sensitivity, which in turn enhances both structural and elastographic image contrast through improved signal-to-noise ratio (SNR) [23,24]. In practice, however, the implementation of high-resolution structural and elastographic imaging in the visible range is challenged by factors such as stronger absorption by specific pigments and the high relative intensity noise (RIN) of commonly used supercontinuum light sources [25]. While the first factor can be considered negligible in the cornea due to its low absorption coefficients [26], the impact of RIN must be assessed and mitigated through appropriate system design and parameter optimization.

In this paper, we present the first demonstration integrating a custom-built vis-OCT prototype system with elastographic imaging protocols, to analyze pulsatile and oscillatory motion in corneal tissue. Our custom setup leverages the high resolution and scattering properties of visible light for structural imaging. In combination with the phase-sensitivity provided by the system, we showcase the system’s performance to detect nanometer-scale tissue motion induced by both, physiological processes, such as the heartbeat dependent fluctuations of the intraocular pressure, and active air-puff-based stimulation techniques. In the following sections, we describe the hardware and software developed for potential active and passive OCE imaging applications in the murine cornea, present measurements of important system performance parameters, and demonstrate in-vivo data obtained without external stimulus as well as using an air puff to excite tissue deformations, underscoring the potential of this approach for longitudinal in-vivo studies of optical and mechanical tissue parameters.

## 2. Materials and methods

### 2.1. Optical setup and imaging protocols

**Fig. 1.**
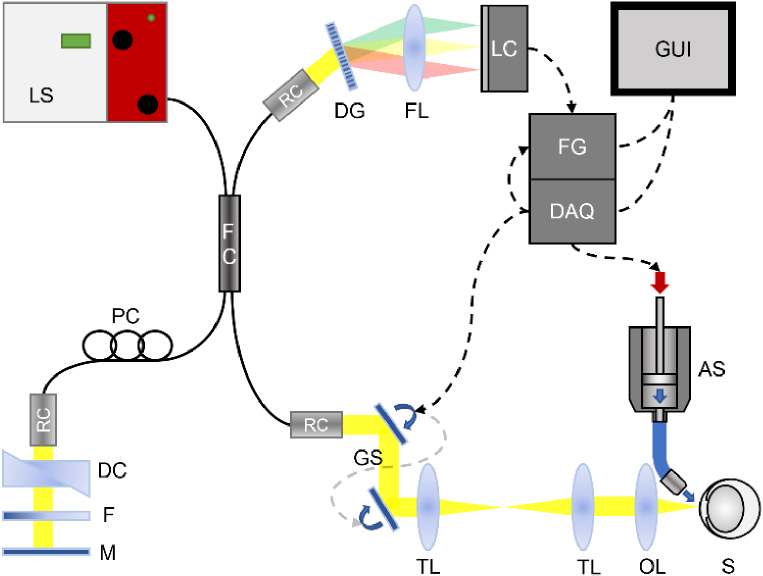
Fiber-based, spectral-domain vis-OCT prototype.: Light source (LS), fiber coupler (FC), polarization control (PC), reflective collimators (RC), dispersion correction (DC), neutral density filter (F), mirror (M), galvanometric scanners (GS), telescopic lenses (TL), objective lens (OL), sample (S), diffraction grating (DG), focus lens (FL), line scan camera (LC), frame grabber (FG), data acquisition card (DAQ), graphical user interface (GUI) and air puff stimulator (AS).

The layout of the custom-built vis-OCT system is shown in Fig. 1 and comprises a fiber-based spectral-domain OCT configuration, optimized for corneal microscopic imaging in mice, and includes additional features for mechanical tissue stimulation during the acquisition process. A supercontinuum laser source (SuperK Extreme EXU-6, NKT Photonics, Birkeroed, Denmark) provided a broad spectrum ranging from 425 to 2400 nm. By using a tunable filter unit (SuperK VARIA, NKT Photonics, Birkeroed, Denmark), the broadband spectrum was truncated to match the wavelength ranges compatible with the fiber coupler and spectrometer. This allowed the OCT system to operate with a spectral bandwidth of 90 nm, centered at approximately 610 nm.

The system is based on a Michelson interferometer design, in which light from the source is split by a 50:50 fiber coupler and directed towards the sample or reference arm. In both arms a reflective collimator (RC02SMA-F01, Thorlabs Inc., Newton, NJ, USA) was used to collimate the light emitted from the fiber tip. The collimated beam in the sample arm was first reflected by a pair of galvo mirrors for 2D scanning (GVS002, Thorlabs Inc., Newton, NJ, USA). A 1:1 telescope was used to project the scanner’s pivot point into the entrance pupil plane of the microscopic objective lens (UPLANFL N 4X, Olympus, Tokyo, Japan). The scanned focus was located on the front surface of the cornea with a working distance of ∼17 mm from the front of the objective lens. In the focal plane, a maximum power of 356 µW was measured. Using a 2 mm spot size, a significantly smaller beam diameter than the objective’s entrance pupil diameter of 11.7 mm was used. This choice was made intentionally to account for beam scanning and to decrease the effective numerical aperture (NA) and expand the system’s depth of focus and lateral resolution in order to be less sensitive to out-of-plane motion artifacts during the acquisition. In the reference arm, a movable mirror on a translational stage was used for adjusting the depth position of the coherence gate. Additionally, a variable neutral density filter served as an intensity regulator and prism pairs were added and adjusted in their thickness to compensate for chromatic dispersion. Backscattered light from the sample and reference arms was interfered, again collimated by a reflective collimator (RC04SMA-F01, Thorlabs Inc., Newton, NJ, USA), and eventually analyzed in the spectrometer arm, where a diffraction grating with 1800 lines/mm was used to split the light into its spectral components. A Planar T* 1.4/85 lens (Carl Zeiss AG, Oberkochen, Germany) was used to project the dispersed spectral line onto a high-speed CMOS camera (VL-4k7C-M200l-2, Vieworks Co., Ltd., Anyang, Republic of Korea), capable of resolving interference fringes at maximum line rate of 83 kHz with a quantum efficiency of ∼70%. The camera was connected to a NI-1437 frame grabber (National Instruments Corp., Austin, TX, United States) and read out at 82 kHz by custom-written LabVIEW software.

Scanning protocols were implemented for different imaging scenarios, ranging from BM-scans for passive protocols, where phase images were subtracted in consecutive frames/B-scans, to reveal physiologically induced motion locally resolved in the corneal layers, to M-scans, for the evaluation of faster vibrations at only one lateral location at the cornea, generated by air-puff stimulation. The specific protocols are summarized in Table 1. Scanning, image acquisition and stimulation were synchronized using the NI-PCI-6232 multifunctional I/O device (National Instruments, Austin, TX, United States). An experimental software prototype was implemented in LabVIEW code, comprising of a real-time visualization of structural OCT data, image acquisition and saving options of raw spectral data.

**Table 1.** Scan protocol specifications.

	M-scan protocol	BM-scan protocol
**Experiment**	Active stimulation	Active/Passive stimulation
**Lateral scanning**	No	Yes
**Lateral scan range**	5.5 µm	0.95 mm
**Number of A-scans per frame**	61,200	500
**Number of B-scans repetitions**	-	500
**Total acquisition time**	0.75 s	3.66 s

The vis-OCT system was equipped with an air puff stimulation unit of a commercial device (XPert NCT; Reichert Inc., USA). Originally designed for dynamic contour tonometry in humans, adjustments were necessary to facilitate a more delicate stimulation of the murine cornea. To induce micro-scale displacements within the corneal tissue parallel to imaging, we connected the air jet outlet with a flexible nozzle that was positioned perpendicular to the corneal surface (Fig. 1). The nozzle’s output diameter was 1.5 mm and the angle between imaging and stimulation axis was approximately 30°. The stimulation trigger was synchronized with the acquisition start and sent after a predefined time delay to activate the air puff generation. For M-scan imaging, a time delay of 50 ms was chosen to enable a baseline measurement before stimulation. The air-puff stimulation lasted for a duration of ∼20 ms.

### 2.2. Processing pipelines for motion detection in the cornea

Standard spectral-domain OCT image pre-processing was implemented to obtain the OCT images from spectral raw data. In more detail, the LabVIEW based pre-processing pipeline included a background subtraction, numerical dispersion compensation and remapping of the spectral data to equidistant sampling in k-space, finally followed by a Fourier transformation to reconstruct the complex OCT signal. Amplitude- and phase images were saved and served as the input for further post-processing steps.

Due to a relatively slow B-scan repetition of 136.7 Hz, any bulk motion artifacts in the BM-scans acquired from the living mouse, breathing or other involuntary movement sometimes caused strong motion artifacts. These could strongly impede phase-based displacement measurements. While out-of-plane motion could only be minimized by finding an optimally centered position of the cornea when scanned with the predefined protocol, transverse and axial bulk motion was compensated in postprocessing by the implementation of a customized auto-registration process. For this purpose, we used consecutive intensity B-scans and estimated the geometric transformation that aligned these two images and thus corrected for axial and transverse shifts on a pixel level.

**Fig. 2.**
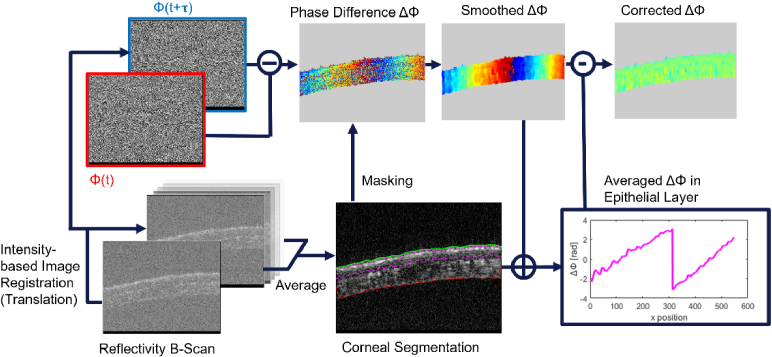
Image processing pipeline for extraction of stromal pulsatile motion *in vivo*: includes intensity-based image registration to reduce inter-frame bulk motion in transverse and axial direction, corneal segmentation for masking and epithelial layer extraction. Phase images are subtracted, smoothed. Sub-pixel bulk motion in the epithelial layer is subtracted to reveal relative motion in the corrected phase difference image.

Axial displacements in the BM-mode data were calculated by complex phase subtraction of consecutive B-scans, resulting in a two-dimensional phase difference B-scan. After convolution with a Gaussian kernel to reduce noise in the masked phase difference image, sub-pixel bulk motion artifacts had to be removed by further processing steps. Motion amplitudes of a thin layer in the corneal epithelium served as a reference and were subtracted from the motion signal in the whole cornea. Thereby, displacements in the cornea were revealed in reference to the corneal epithelium, and thus could be used to quantify stromal compression and expansion induced by the heart-beat dependent intraocular pressure (IOP) fluctuations. Figure 2 provides a flow chart visualization of these processing steps.

The axial displacement between successive frames was calculated from the corrected phase difference images using the formula

(1) $$\Delta d=\frac{\lambda_{0}\Delta \phi }{4\pi n},$$

where Δ*d* is the axial displacement in nanometers, λ_0_ is the central wavelength of the OCT system in nanometers, Δ*ϕ* is the phase difference in radians, and n stands for the refractive index of corneal tissue which was assumed as n = 1.35.

To extract consistent motion signals over time and ensure a more reliable displacement measurement, additional processing steps were implemented. First, given by stronger reflectivity in that central region compared to the periphery of the corneal scan, a region of interest (ROI) was manually selected, to minimize the impact of peripheral signal degradation on the extracted motion profiles. Displacement maps extracted from the ROI were flattened and averaged along the lateral scanning dimension, to generate a one-dimensional depth displacement profile for the central cornea in each frame. To further refine the signal and suppress high-frequency components unrelated to slow physiological motion, Gaussian filtering was applied over time and depth.

In the M-scan mode for tracking of air-puff induced oscillations, lateral scanning was deactivated. Nevertheless, the set of A-scans was pre-processed similarly to the BM-scans, with the only difference of measuring one background depth profile beforehand instead of calculating it from each single B-scan separately. To properly resolve faster oscillations, induced by the air puff stimulator, phase differences were computed A-scan-wise over the whole M-scan, resulting in a higher temporal resolution for displacement measurements. However, the BM-scan mode was still used for aligning the mouse eye, so that the M-scan could be acquired at a suitable position, preferably in the center of the cornea.

In both cases, i.e. for passive and active protocols, a time-frequency analysis was performed. Temporal variations of micro-displacements were charted over time and oscillation frequencies were computed by Fourier transforming the time signal.

### 2.3. Animal preparation and imaging procedure

As a proof of concept, the corneas of healthy C57BL/6J mice (Charles River, 34 weeks old, male) were investigated, by using either the passive or active approach. While the air-puff experiment was performed *ex vivo* on mounted formalin fixed mouse eyes, the passive approach required a predefined imaging protocol for *in vivo* experimentation: prior to experimental imaging, anaesthesia was induced in a chamber ventilated with isoflurane vaporized in oxygen at 4% concentration for a total incubation time of 4 minutes. Then, an anaesthetic mixture of medetomidine (0.3 mg/kg body weight), midazolam (1.0 mg/kg), fentanyl (0.03 mg/kg) and ketamine (10.0 mg/kg) was applied as an intraperitoneal injection to ensure anaesthesia to last throughout the experimental imaging procedure, without the need for continuous administration of isoflurane. Heat pads were used to maintain the body temperature. In between image acquisitions, artificial teardrops (Oculotect artificial eye drops, Théa Pharma GmbH, Germany) were used to keep the eyes moisturized. The entire duration of the image session took about 30 minutes, starting from pre-incubation. All animal experiments were conducted in accordance with the ARVO Statement for the Use of Animals in Ophthalmic and Vision Research, and the applied protocols were approved by the animal ethics committee of the Medical University of Vienna and the Austrian Ministry of Education, Science, and Research (BMBWF/66.009/0272-V/3b/2019).

## 3. Results

### 3.1. System specifications

**Fig. 3.**
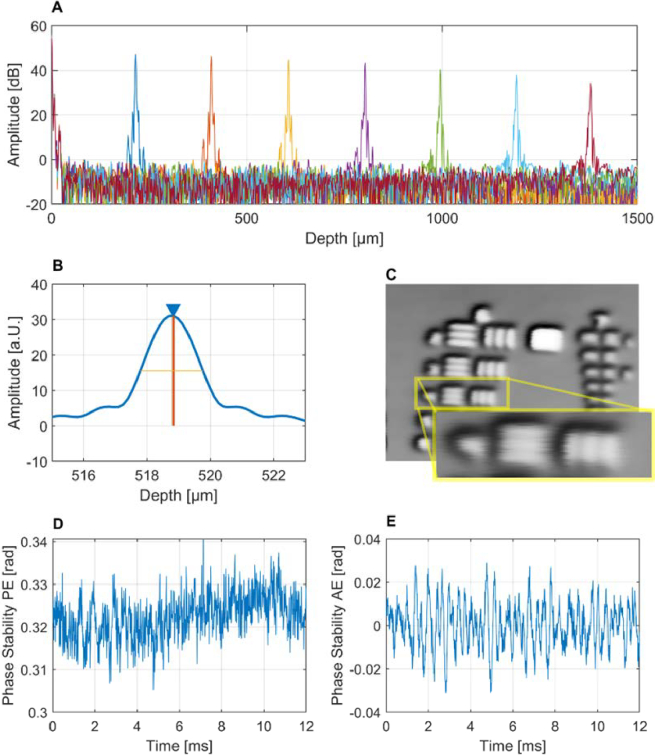
System specifications of the vis-OCT prototype. (A) The axial SNR roll-off was 10.5 dB/mm. (B) The axial resolution was 2.0 µm. (C) The lateral resolution was estimated as 5.5 µm using a reflective resolution test target. (D) The phase noise for passive protocols was 5.2 mrad over 12 ms. (E) The phase noise for active protocols was 10.6 mrad over 12 ms (SNR_coverslip_ = 57 dB).

Figure 3 shows the specification measurements to evaluate the system’s performance. The axial sensitivity decay was derived from the depth-dependent signal amplitudes shown in Fig. 3(A). By analyzing the maximum signal amplitudes and their corresponding peak positions obtained through axial stepping of the reference mirror, the sensitivity roll-off was calculated to be approximately 10 dB/mm. Figure 3(B) shows the axial point-spread-function of a mirror placed in the focus position of the sample beam path, indicating a maximum resolution (FWHM, yellow bar) of 2.0 µm in corneal tissue. With the same mirror configuration, a sensitivity of 86 dB was measured at a line rate of 82 kHz and maximum sample illumination power of 356 µW. The lateral resolution δx was estimated to be ∼5.5 µm by visually resolving features up to group 6, element 4 on a USAF 1951 resolution target (Fig. 3(C)). From this lateral resolution, the objective’s effective NA of 0.067 was inferred using the diffraction-limited relation NA = 0.61λ/δx at λ = 610 nm. The corresponding depth of focus was then calculated as DOF = λ/(2 NA^2^) ≈ 67 μm. Furthermore, we evaluated the phase stability of the system by placing a glass coverslip centered in the focus position and measuring the phase variation along the front- and back surface over a time period of 12 ms. Two phase noise values were computed from this data set, according to different data processing steps in the protocols used for elastrographic imaging. According to the BM-scan protocol, we subtracted absolute phase values of the back surface from those of the front surface. The resulting signal in Fig. 3(D) represents a relative measure of phase variation, which depends inverse proportionally on the local SNR. Expressed as the standard deviation of the phase difference signal shown in the phase stability plot, a phase noise of 5.23 mrad was found at a corresponding SNR of 57 dB. Similarly, the signal shown in Fig. 3(E) was computed for the data sets acquired with the M-scan protocol, by phase subtraction of consecutive A-scans over time along the front surface only, leading to a phase noise value of 10.6 mrad. Both methods minimized the effect of global, environmentally-induced fluctuations of the phase. All in all, this setup allowed the simultaneous acquisition of high-resolution structural intensity and functional data of tissue motion, whether actively induced or physiological.

### 3.2. High-resolution vis-OCT

**Fig. 4.**
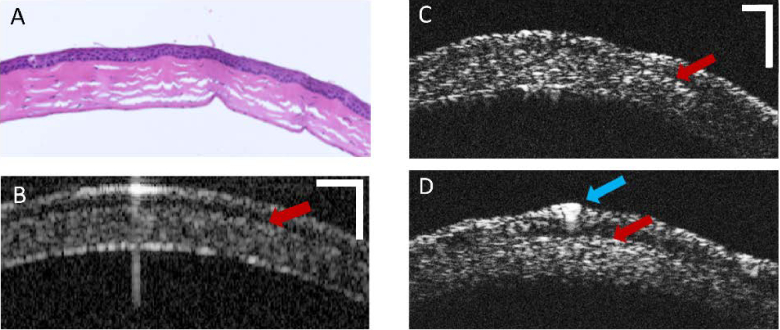
The murine cornea analyzed with different modalities: (A) Histological section of the cornea. (B) Corneal reflectivity B-scan in conventional NIR-SD-OCT compared to exemplary vis-OCT B-scans of murine corneas in peripheral (C) and central (D) location, highlighting improved resolution in vis-OCT. Red arrows: transition zone with less signal strength between epithelium and stroma. Blue arrow: increased reflectivity in the central cornea. Scale bars: 100 µm.

*In vivo* imaging of anterior sections in mouse eyes was performed using the prototype. In Fig. 4, single B-scans are compared with previously reported data [27].The images shown in Figs. 4(C-D) reveal corneal tissue structures with a resolution and contrast, enabling distinct layer-specific structural differentiation within the cornea. Red arrows indicate a transitional zone between epithelium and stroma, located around the Bowman’s layer. Signal losses were observed in the peripheral cornea due to reduced backscattered light in this region, with decreasing contrast in deeper layers. The blue arrow highlights a stronger back reflection in the central cornea, possibly a scar induced by corneal scratching. A pronounced signal heterogeneity corresponds to the underlying cellular structure, comprising keratocytes and collagen fibers, across the entire cornea and especially in the stromal region. Figure 4(B) shows a comparative B-scan of a murine cornea obtained with a SD-OCT system operating at a central wavelength of 837 nm and an axial resolution of 5.6 µm in corneal tissue [27]. A comparative histological section is provided in Fig. 4(A).

### 3.3. Passive investigation of pulsatile, physiological motion

**Fig. 5.**
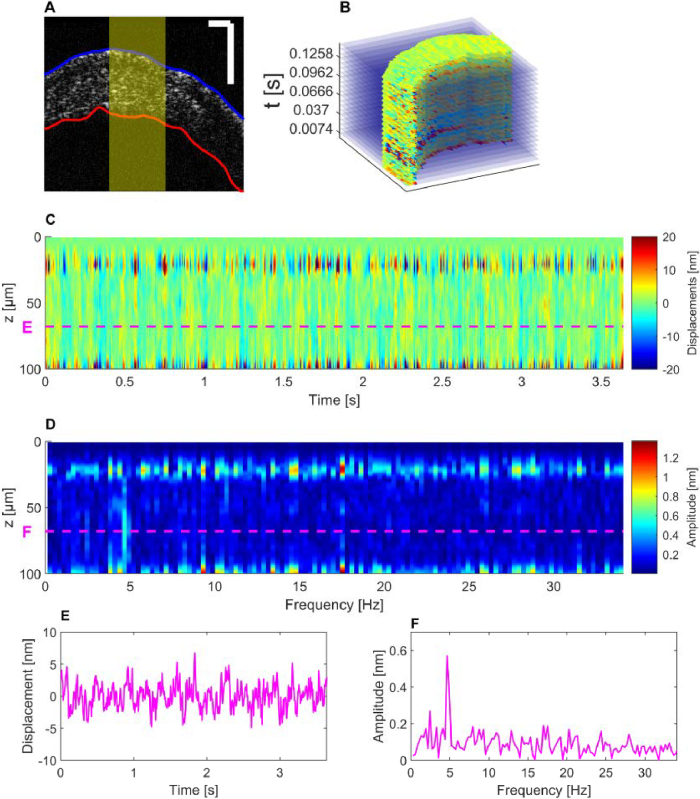
Heartbeat-induced pulsation frequency in central stroma revealed by in-vivo vis-OCT. (A) B-scan with segmented mouse cornea. Scale bar: 100 µm. (B) Stack of processed displacement maps. (C) Averaged displacement in the central cornea highlighted in yellow in panel (A) along time shows the pulsatile behavior in the stromal layer. (D) Frequency analysis shows frequency peak at 4.6 Hz. Panels (E) and (F) display the pulsatile motion and frequency at a depth of 68 µm within the cornea.

Based on the BM-scan protocol, a set of 500 consecutive B-scans was acquired in the cornea of a live mouse, with complex OCT images serving as input for the processing pipeline. This resulted in 499 displacement maps generated over a total acquisition period of 3.66 seconds. The selected ROI in the central cornea is highlighted by a yellow square in the intensity B-scan in Fig. 5(A), while Fig. 5(B) reveals spatially resolved motion inside the cornea monitored over time. After corneal flattening and transverse averaging, a depth resolved temporal displacement profile of the cornea was obtained, as shown in Fig. 5(C), where decorrelation artifacts in several frames could be successfully compensated by the introduced filter methods. The signal was clearly dominated by a periodic oscillation in the stroma. The frequency analysis (Fig. 5(D)) indicated a prominent peak around 4.6 Hz, which confirmed the periodic character of motion patterns, specifically in the stromal layer of the cornea, where signal intensity was strongest besides the anterior part of the epithelium. However, in regions with lower intensity, e.g. around Bowman’s layer between epithelium and stroma, the resulting rather noisy phase signal did not allow a reliable motion measurement. Figures 5(E-F) show the oscillatory motion and its frequency characterization at a depth of 68 µm. Under the assumption of a sinusoidal signal, the amplitude was estimated as $A=\sqrt{2}\cdot \sigma =2.7nm$ , with *σ* as the standard deviation computed over the oscillatory displacement signal in Fig. 5(E). The frequency peaks at 2.5 Hz and 4.6 Hz, seen in Fig. 5(F), clearly emphasized the periodic character of physiological induced compression and expansion of the stroma in the nanoscale range.

### 3.4. Air-puff induced nanoscale oscillations

Similar to previous methods used in passive conditions, we also implemented a time-frequency analysis, investigating nanoscale motion induced by active air-puff stimulation on a mounted ex-vivo mouse eye.

**Fig. 6.**
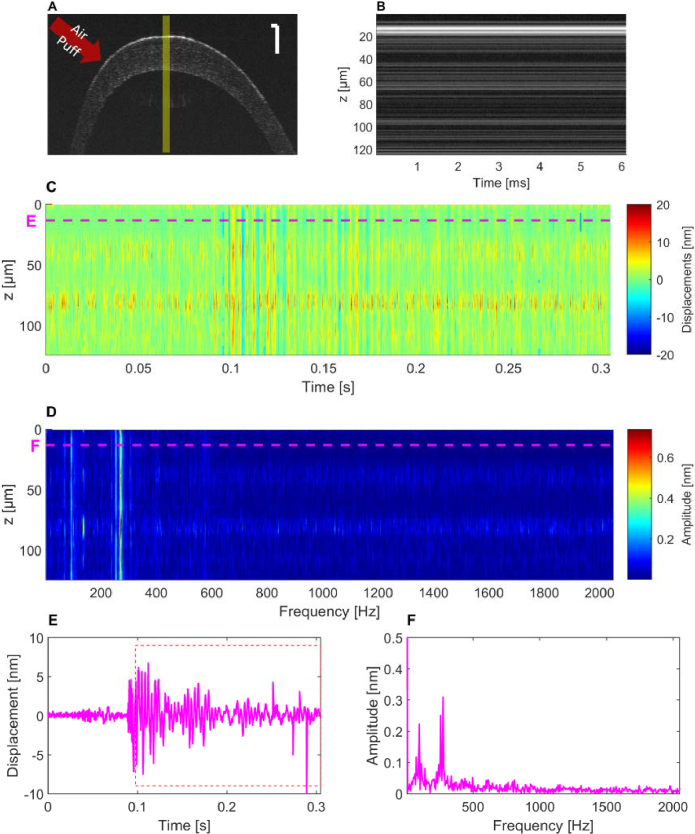
Air-puff-induced oscillation frequencies in the *ex vivo* mouse eye extracted from vis-OCT M-scans. (A) B-scan of mouse cornea with M-scan position highlighted in yellow. Scale bar: 100 µm. (B) M-scan intensity profile acquired at the central cornea. (C) Depth-resolved displacement signal extracted via A-scan-wise phase subtraction shows pronounced oscillating waves in corneal layers. (D) Frequency analysis shows strongest frequency peaks at 252 Hz and 269 Hz in the epithelium. Panel (E) shows the pulsatile motion in the epithelial layer at a depth of 13.3 µm from the corneal surface and (F) the corresponding frequency spectrum, computed via FFT from windowed motion signal (red dashed box).

Figure 6(A) displays an exemplary B-scan, in which the M-scan position and the peripheral location of the air-puff stimulation are marked by a yellow rectangle and a red-arrow, respectively. Figure 6(B) demonstrates a temporal window of 6 ms in the intensity M-scan. While this sequence contains the maximum stimulation amplitudes registered in the corresponding phase signal, no oscillations were resolvable in the OCT intensity image. However, an oscillatory movement can be observed in the displacement signal all over the cornea after stimulation, but specifically in the epithelial layer with highest signal strength and thus the strongest phase stability (Fig. 6(C)). The displacement signal, presented in Fig. 6(E) shows a damped oscillation in the epithelial layer, with its corresponding frequency spectrum in Fig. 6(F), extracted from a temporal window, marked as a red dashed box in (E), over a duration of 0.2s. The spectrum indicates most prominent peaks around 274.9 Hz, 255.6 Hz and 96.5 Hz. In this layer, a maximum displacement amplitude of -6.9 nm was detected approximately 10 ms after initialization of the air-puff, where also the pressure achieved its maximum, as indicated in earlier reports on the stimulation device [28]. To assess the repeatability of oscillation frequency measurements induced by air-puff stimulation, four ex vivo murine eyes were imaged using the M-scan protocol. The most prominent frequency peaks observed were 255.6 Hz in three samples and 274.9 Hz in one sample, resulting in a mean dominant frequency of 260.4 ± 8.8 Hz.

## 4. Discussion and conclusion

In this work we investigated the corneal motion response to either a passive or active stimulation on the nanoscale level. First, physiologically induced compression and expansion in the murine cornea was revealed by a combination of vis-OCT with the specified phase image processing pipeline. Motion inside the cornea was measured relative to the epithelium, and was best detectable in the posterior stroma where both the SNR and the relative motion amplitudes to the epithelium are largest. Here, a strong periodicity was revealed, with two distinct frequency peaks. The dominant peak at approximately 4.6 Hz falls within the typical heart rate range of an anesthetized mouse [26] and is therefore likely associated with heartbeat-induced IOP modulation. A second frequency peak was observed at 2.5 Hz, which closely aligns with residual bulk motion measured at 2.2 Hz, suggesting that breathing-related bulk motion was not fully corrected by the intensity-based image registration prior to phase-sensitive analysis, or alternatively, that breathing contributes an additional compressive component within the cornea, alongside the cardiac-induced displacement at 4.6 Hz. This interpretation is further supported by the observation that displacement amplitudes at these specific frequencies increase steadily from noise levels at the epithelial reference layer to a maximum in the central stroma. Similar observations were made by Nair et al, who reported a physiologically induced strain gradient across the whole cornea in rabbits using in vivo heart-beat OCE, a contact-based elastography approach [14]. In contrast, our measurements generally reveal only modest gradients in the anterior cornea and a decline in amplitude from center towards the posterior stroma. This discrepancy can likely be attributed to a combination of technical and biological factors, including the inherently smaller physiological deformation levels stemming from the non-contact approach, which result in lower displacement amplitudes. Additionally, lower signal intensities in the posterior cornea may lead to increased phase noise, causing the frequency “washout” effect, characterized by a broadened and less distinguishable frequency peak observed in this region. With the highest SNR in the central stroma, the average maximum interframe displacement amplitude was measured to be 2.7 nm. This finding may complement the results reported by Peng Li et al., who investigated ocular pulse-induced displacements of the murine lens relative to the cornea using phase-sensitive OCT [29]. In their study, an average lens velocity of 10.3 μm/s was reported. When scaled to our system’s B-scan acquisition rate of 136.7 Hz, this corresponds to an approximate interframe displacement of 75.3 nm.

Future improvements could aim toward whole-eye acquisitions to assess ocular nanoscale dynamics across the entire depth of the eye. Additionally, we seek to achieve spatially resolved visualization of displacement amplitudes within the cornea, similar to prior demonstrations in other anterior segment structures. For instance, Xin et al. used phase-sensitive OCT to quantify the pulsatile, phase-synchronous motion of the trabecular meshwork (TM) in response to aqueous humor outflow in healthy human subjects, reporting high repeatability and a clear correlation with the cardiac cycle [30]. While such studies share the common physiological driver of pulsatile ocular blood flow, it is essential to acknowledge the differences in anatomical context and mechanical response. Structural responses, such as TM deformation or corneal compression, are influenced by local biomechanical properties and vary with tissue architecture. In our case, corneal compression could be observed by averaging the centrally located displacement signal in each B-scan; however, lateral heterogeneity in signal quality limits the analysis of spatially coherent displacement patterns. This suggests that even though the detected motion amplitudes were shown to exceed the system’s motion detection sensitivity limit of 0.19 nm, which is constrained by its phase stability, other factors may have influenced capability of a laterally resolved motion measurement as well. Based on the *in vivo* imaging conditions, breathing and other involuntary movements could have led to out-of-plane motion, which potentially contributed to decorrelation artifacts, that cannot be corrected by translational image registration. To enable and improve elastographic measurements in future vis-OCT implementations, recently proposed methods to improve the signal quality may be taken into consideration. Rubinoff et al. showed that vis-OCT implemented with as dual-channel balanced detection could lead to significant improvements of the SNR [31]. Considering the dependence of phase difference measurements on SNR [23], also phase-based OCE measurements are likely to benefit from a balanced detection scheme. Furthermore, Lan et al. demonstrated that the phase stability of an OCE system may be improved by switching from the standard implementation of an SD-OCT configuration including separated sample and reference arms to a common-path system design where both arms are combined into one [8]. While their results showed the improvement of phase stability based on an OCT system working in the NIR range, the principle could be extended to vis-OCT as well.

In addition to the passive approach, we performed a displacement measurement of an actively induced stimulation. Here, nanoscale oscillatory motion was revealed by phase subtraction of consecutive A-scans acquired continuously in the center of the cornea after air-puff stimulation. The most dominant frequency peaks of the damped oscillatory signal were observed at 255.6 Hz in three samples and 274.9 Hz in one sample. While the observed frequency variation across samples indicates a reasonable level of repeatability, the small sample size limits statistical significance. Additionally, the frequency resolution is constrained by the 4.82 Hz sampling interval of the M-scan mode, reducing the precision of frequency estimation. The short temporal window due to rapid damping of the oscillatory signal further restricts spectral resolution. Future studies will address these limitations by increasing sample size and incorporating repeated stimulations to extend the observation window. This could enhance frequency resolution and enable more advanced time-frequency analysis to better characterize corneal viscoelastic behavior.

Assuming an underdamped oscillatory response, consistent with the gradual amplitude decay observed following air-puff stimulation, the natural frequencies are expected to lie close to the dominant spectral peaks. The measured frequencies of 255.6 Hz and 274.9 Hz fall within the range of natural corneal frequencies previously reported in vivo in human eyes (234–277 Hz) [32]. This correspondence supports the physiological plausibility of the observed oscillations, despite the low sample size in our study. However, the potential impact of species-specific anatomical differences warrants further investigation in larger cohorts.

Several improvements of the stimulation technique and the applied scanning protocol can be considered to further refine the measurement accuracy and repeatability of our technique. In particular, when compared with previous work on the corneal response [32,33], it was noticeable that instead of having an initial maximum displacement, the amplitudes in our results continuously increased over multiple periods. As mentioned earlier, the stimulation device was adapted from the air-puff system described by Jiménez-Villar et al [28]. It was originally designed to generate air pulses strong enough to macroscopically deform the human cornea, delivering forces in the milli-Newton range and lasting several milliseconds. The adaption for imaging of mouse eyes here, could be improved by the integration of more delicate stimulation techniques, such as the flow-controlled air puff generation with forces in the µN range as previously introduced by Detrez et al. for instance [34,35]. Furthermore, shorter air-puff durations could lead to improved tissue responses [36]. Additionally, instead of limiting the evaluation to a single position based on an M-scan in the central cornea, the extension with an MB-scan protocol could further be of interest for investigations of the corneal response temporally resolved in two or even three spatial dimensions.

In summary, we have shown that similar to phase-sensitive OCT in the NIR, vis-OCT can be used for measurement of displacements on the nanometer scale, as demonstrated here in the murine cornea. These measurements may serve as a relative indicator of tissue elasticity or could provide the necessary information to eventually estimate absolute elastography parameters such as the strain or natural frequency. To validate a quantitative mechanical assessment based on displacement measurements, further experiments are required to demonstrate the system’s reliability in detecting variations in elasticities, for example by testing different engineered tissue samples. Additionally, the elastography approach could be evaluated in a preclinical context by analyzing pulsatile amplitudes in crosslinked versus healthy mouse corneas. This opens up a “biomechanical” contrast channel, complementing the high-resolution structural imaging capabilities of vis-OCT. The combination of high-resolution structural imaging with elastographic measurements provided by vis-OCT may ultimately contribute to improved diagnostic capabilities and therapeutic monitoring in ophthalmic OCT. Future work based on the research presented here may focus on the development of a fully non-invasive method for studying structure-related corneal biomechanics in preclinical settings.

## Data Availability

Data underlying the results presented in this paper are not publicly available at this time but may be obtained from the authors upon reasonable request.
